# Cell surface localization of importin α1/KPNA2 affects cancer cell proliferation by regulating FGF1 signalling

**DOI:** 10.1038/srep21410

**Published:** 2016-02-18

**Authors:** Kohji Yamada, Yoichi Miyamoto, Akira Tsujii, Tetsuji Moriyama, Yudai Ikuno, Takashi Shiromizu, Satoshi Serada, Minoru Fujimoto, Takeshi Tomonaga, Tetsuji Naka, Yoshihiro Yoneda, Masahiro Oka

**Affiliations:** 1 Laboratory of Nuclear Transport Dynamics, National Institutes of Biomedical Innovation, Health and Nutrition, 7-6-8 Saito-Asagi, Ibaraki, Osaka 567-0085, Japan; 2 Department of Genetics, Graduate School of Medicine, Osaka University, 1-3 Yamadaoka, Suita, Osaka 565-0871, Japan; 3 Laboratory of Proteome Research, National Institutes of Biomedical Innovation, Health and Nutrition, 7-6-8 Saito-Asagi, Ibaraki, Osaka 567-0085, Japan; 4 Laboratory of Immune Signal, National Institutes of Biomedical Innovation, Health and Nutrition, 7-6-8 Saito-Asagi, Ibaraki, Osaka 567-0085, Japan; 5 National Institutes of Biomedical Innovation, Health and Nutrition, 7-6-8 Saito-Asagi, Ibaraki, Osaka 567-0085, Japan; 6 Laboratory of Biomedical Innovation, Graduate School of Pharmaceutical Sciences, Osaka University, Suita, Osaka 565-0871, National Institutes of Biomedical Innovation, Health and Nutrition, Ibaraki, Osaka 567-0085, Japan.

## Abstract

Importin α1 is involved in nuclear import as a receptor for proteins with a classical nuclear localization signal (cNLS). Here, we report that importin α1 is localized to the cell surface in several cancer cell lines and detected in their cultured medium. We also found that exogenously added importin α1 is associated with the cell membrane via interaction with heparan sulfate. Furthermore, we revealed that the cell surface importin α1 recognizes cNLS-containing substrates. More particularly, importin α1 bound directly to FGF1 and FGF2, secreted cNLS-containing growth factors, and addition of exogenous importin α1 enhanced the activation of ERK1/2, downstream targets of FGF1 signalling, in FGF1-stimulated cancer cells. Additionally, anti-importin α1 antibody treatment suppressed the importin α1−FGF1 complex formation and ERK1/2 activation, resulting in decreased cell growth. This study provides novel evidence that functional importin α1 is located at the cell surface, where it accelerates the proliferation of cancer cells.

Nuclear−cytoplasmic transport of karyophilic proteins is a process that is conserved across species. In this process, signal sequences of cargo proteins, including classical nuclear localization signals (cNLSs), are recognized by transport factors to allow the cargo proteins to pass through the nuclear pore complex (NPC) between the cytoplasm and the nucleus^1^,^2^,^3^,^4^,^5^. Among these transport factors is importin α, which was characterized as a cNLS receptor that mediates the nuclear transport of divergent substrates containing the cNLS. In the cytoplasm, importin α recognizes cargo containing a cNLS, followed by association with importin β that is essential for association with the NPC, and in this way, the cNLS-cargo/importin α/importin β ternary complex is translocated from the cytoplasm to the nucleus via the NPC^3^,^4^,^5^. In the nucleus, dissociation of the complex and concurrent release of importin α and the cargo occur because of binding of a GTP-bound form of a small GTP-binding protein, Ran (RanGTP), to importin β. Thereafter, detached importin α forms a distinct complex in the nucleus with the cellular apoptosis susceptibility protein (CAS, also referred to as CSE1L), in conjunction with RanGTP, and is recycled back to the cytoplasm. Thus, it has been demonstrated that importin α functions in the nuclear−cytoplasmic transport within cells^3^,^4^,^5^.

In humans, seven subtypes of importin α, which show different tissue-specific expression patterns and distinct cargo specificities, have been identified to date^3^,^6^,^7^,^8^. Importin α1, also referred to as karyopherin alpha (KPNA) 2, is one of the importin α subtypes, and is highly expressed and well-characterized in cultured cells in general (such as HeLa cells), ES cells, and germ cell lines^9^,^10^. In these cells, importin α1 has been implicated in a wide variety of physiological cellular processes, including cell differentiation, spermatogenesis, as well as in human diseases^10^,^11^,^12^.

Furthermore, many studies have recently reported that importin α1 is highly expressed in diverse types of cancers, including breast cancer, hepatocellular carcinoma, lung cancer, melanoma, and ovarian cancer^13^,^14^,^15^,^16^. Such aberrant importin α1 expression is often correlated with an adverse outcome in patients^13^. Although subcellular localization of importin α1 is diffuse throughout cells^17^, it has been shown that importin α1 is also detected in the sera of lung cancer patients^18^. However, it is still poorly understood how importin α1 is involved in cancerous processes.

In this study, using a combination of flow cytometric, biochemical, and confocal microscopic approaches, we show for the first time that importin α1 is localized to the cell surface in several human cancer cell lines. Furthermore, we found that importin α1 at the cell surface is associated with a growth factor, FGF1, thereby enhancing its signalling pathway and accelerating the proliferation of cancer cells. This is the first evidence showing that proteins that ordinarily function within cells can localize to the cell surface where they participate in novel physiological activities.

## Results

### Importin α1 is localized to the cell surface in some cancer cell lines

**Figure 1:**
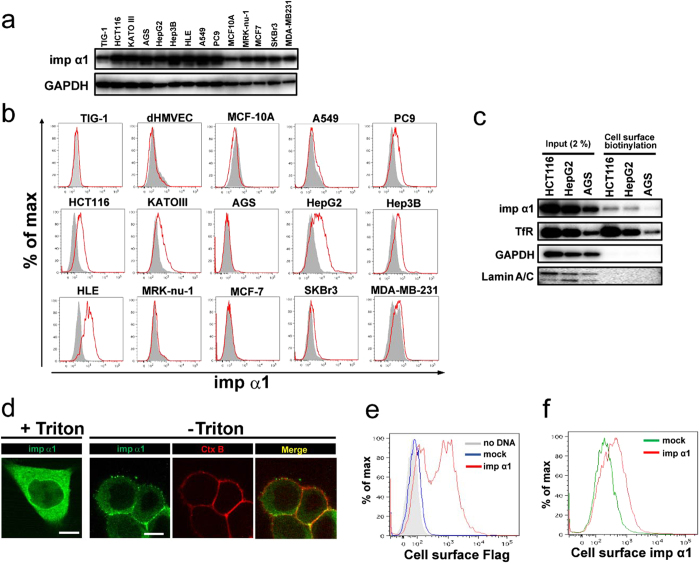
Importin α1 is localized at the cell surface of several cancer cell lines. (**a**) Total cell lysates of each cell line were subjected to an immunoblot analysis. GAPDH was used as a control. (**b**) Expression of importin α1 on the cell surface of several cancer cell lines, normal cell lines, and primary cells. Gray area, isotype control antibody (Ab). (**c**) Cell surface proteins on HCT116 cells, HepG2 cells, and AGS cells were biotinylated using NHS-biotin. Extracts of each fraction were pulled-down with neutravidin, and subjected to immunoblot analysis. Transferrin receptor (TfR) was used as a cell surface marker. Both GAPDH and Lamin A/C were used as a non-cell surface marker. (**d**) For conventional immunostaining, HCT116 cells were fixed, and subsequently permeabilized with 0.1% Triton X-100 for 5 min. For immunostaining under non-permeabilized conditions, living HCT116 cells were pretreated with extracellular tracer CtxB for 1 h on ice, fixed, and stained for importin α1. Representative cells are shown. Merged images were generated from Alexa488 staining (importin α1) and Alexa647 staining (CtxB). Co-localization is indicated in yellow. Scale bars: 10 μm. (**e**) Living HCT116 cells transfected with the empty vector (mock; blue line) or Flag-tagged importin α1 expression vector (red line) were stained with anti-Flag antibody, and subjected to flow cytometric analysis. (**f**) Living HCT116 cells transfected with the empty vector (mock; green line) or Flag-tagged importin α1 expression vector (red line) were stained with anti-importin α1 antibody, and subjected to flow cytometric analysis.

Recently, we performed cell-based proteomic experiments using human vascular endothelial cells to screen for cell surface protein targets that may be involved in systemic sclerosis^19^. Among this proteomic data, we noticed that importin α1 (Importin subunit alpha-1) was included as a potential cell surface protein^19^ (Supplementary Table S1). Furthermore, we performed another proteomic analysis aimed at novel cell surface marker discovery, by using colon cancer cells and tissues. Membrane fraction proteins that had been separated by homogenization and centrifugation also included importin α1 (Supplementary Table S1). Given that high levels of importin α1 expression have been reported in various types of cancers^13^, we assessed whether importin α1 is actually localized at the cell surface by performing flow cytometric analysis using two different antibodies against importin α1 in several types of cancer cell lines. These included the lung cancer cell lines A549 and PC9, gastric cancer cell lines KATOIII and AGS, the colon cancer cell line HCT116, hepatocellular carcinoma cell lines HepG2, Hep3B, and HLE, breast cancer cell lines MRK-nu-1, MCF-7, SKBr3, and MDA-MB-231. We also analysed three normal cell lines: a human fibroblast cell line TIG-1, human dermal microvascular endothelial cells (dHMVECs), and a normal human mammary epithelial cell line MCF-10A. These cell lines express endogenous importin α1 to some extent (Fig. 1a). As shown in Fig. 1b, we detected cell surface importin α1 in PC9, HCT116, KATOIII, HepG2, Hep3B, and HLE cells, while no signals were detected in A549 and AGS cells, or in the three normal cell lines. More particularly, relatively higher levels of cell surface importin α1 were detected in all hepatocellular carcinoma cell lines. On the other hand, none of the four tested breast cancer cell lines expressed importin α1 at the cell surface, suggesting that the cell surface localization of importin α1 may occur in a cell type-dependent manner. Similar results were obtained using the other anti-importin α1 antibody (Supplementary Fig. S1). Thus, for further studies, we mainly focused on colon cancer and hepatocellular carcinoma cell lines.

To confirm the findings above, we next performed a cell surface biotinylation assay, in which cell surface proteins of HCT116, HepG2, and AGS cells were first biotinylated, and the cell lysates were then incubated with avidin beads to precipitate the cell surface biotinylated proteins, followed by immunoblot analysis. As shown in Fig. 1c, we detected a single band of importin α1 in the biotinylated cell surface fraction of HCT116 and HepG2 cells, whereas faint importin α1 was detected in that of AGS cells.

Furthermore, we attempted to visualize cell surface expression of importin α1 in HCT116 cells using confocal laser scanning microscopy. Intracellular importin α1 showed diffuse localization throughout the cell after detergent-based cell permeabilization after fixation (Fig. 1d, +Triton), as reported previously^17^. In contrast, cell surface staining of importin α1 was clearly observed in non-permeabilized cells, with only faint signals in the cytoplasm (Fig. 1d, −Triton). In addition, we found that importin α1 was co-localized with Cholera toxin B (CtxB), which is a known cell surface marker, confirming that importin α1 was expressed on the cell surface.

To further confirm that importin α1 is localized to the cell surface, HCT116 cells were transiently transfected with the N-terminal Flag-tagged importin α1 expression vector. Flow cytometric analysis using either anti-Flag antibody (N-terminus, Fig. 1e) or anti-importin α1 antibody (which recognizes the C-terminal side, Fig. 1f) both showed positive signals in importin α1-overexpressing cells, indicating that the whole importin α1 molecule is exposed to the extracellular milieu. Of note, the amount of cell surface importin α1 was markedly augmented in HCT116 cells expressing Flag-tagged importin α1, as compared with that in the cells transfected with the control vector (Fig. 1f). Taken together, we concluded that importin α1 is present on the surface of these cancer cells.

### Cell surface importin α1 is functional

**Figure 2:**
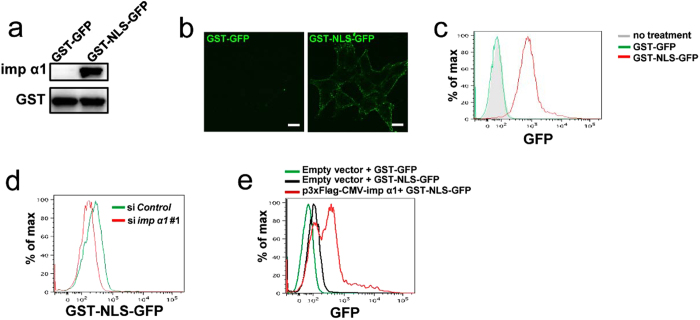
Importin α1 on the cell surface is functional. (**a**) Either GST-GFP or GST-NLS-GFP (50 pmol each) was incubated with 50 pmol of 3 × Flag-tagged importin α1 immobilized on glutathione beads. The bound proteins were analysed using immunoblotting with an anti-importin α1 or an anti-GST antibody, respectively. (**b**) Live HCT116 cells were incubated with GST-GFP or GST-NLS-GFP (1 μg/ml) for 1 h on ice, and we then observed fluorescence after fixation. Representative cells are shown. Scale bars: 10 μm. (**c**) Live HCT116 cells were treated with GST-GFP or GST-NLS-GFP (1 μg/ml) for 1 h on ice, and subjected to a flow cytometric analysis. (**d**) HCT116 cells transfected with control or importin α1 siRNAs, followed by treatment with GST-NLS-GFP (100 ng/ml) for 1 h on ice, and subjected to a flow cytometric analysis. (**e**) HCT116 cells were transfected with the empty vector (mock) or Flag-tagged importin α1 expression vector, followed by treatment with GST-GFP or GST-NLS-GFP (100 ng/ml) for 1 h on ice, and subjected to a flow cytometric analysis.

The predominant feature of cNLS is a sequence comprising one or two short clusters of basic amino acids, such as lysine or arginine. The best characterized NLS is the monopartite type found in the SV40 large T-antigen (PKKKRKV), which is recognized by importin α1. Therefore, next, to address whether the cell surface importin α1 is functional, we examined whether it binds to a typical NLS-containing substrate, the SV40 large T antigen NLS-fused GST and GFP (GST-NLS-GFP), which is known to bind to importin α1^20^ (Fig. 2a). HCT116 cells were incubated with GST-NLS-GFP or GST-GFP (as a negative control). Immunofluorescent analysis revealed GST-NLS-GFP on the cell surface (Fig. 2b). Comparable data was also obtained by flow cytometric analysis using HCT116 cells (Fig. 2c).

Furthermore, to examine whether the association of GST-NLS-GFP with HCT116 cells is dependent on importin α1, we manipulated the cell surface expression levels of importin α1 by knockdown or overexpression procedures. Knockdown of importin α1 by siRNA significantly decreased the amount of cell surface importin α1 (Supplementary Fig. S2), leading to a substantial decrease of GST-NLS-GFP association with the surface of HCT116 cells (Fig. 2d). In contrast, the overexpression of importin α1 resulted in a significant increase in the intensity of GST-NLS-GFP fluorescence on the cell surface (Fig. 2e). These data indicated that the cell surface importin α1 is able to recognize proteins with cNLSs. Collectively, these data indicated that cell surface importin α1 is functional, and not denatured.

### Importin α1 is detected in cultured medium

**Figure 3:**
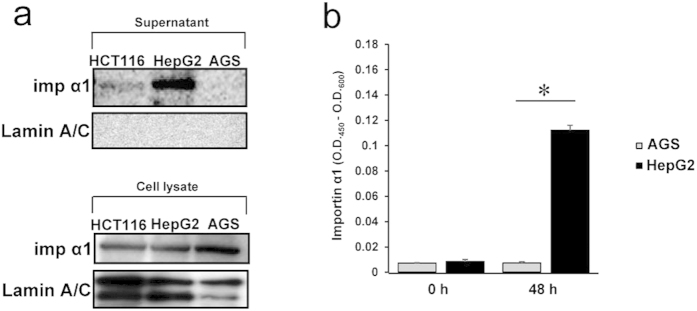
Importin α1 is released into the extracellular milieu by cell surface importin α1-positive cancer cells. (**a**) Subconfluent cancer cells were incubated with fresh medium. After 48 h, supernatants (upper panel) and lysates (lower panel) of cultured cancer cell lines were collected. Immunoblot analysis were performed using antibodies to importin α1 or Lamin A/C (as control). (**b**) Free importin α1 levels in the conditioned medium of cancer cell lines (HepG2 and AGS) were determined using ELISA. Data are means ± SD from three independent experiments. *P < 0.001.

It has previously been reported that importin α1 is detected in the sera of patients with lung cancer^18^. Therefore, we assessed whether importin α1 could be detected in cultured medium of cancer cell lines. After the removal of dead cells and debris by both centrifugation and filtration, the importin α1 protein levels in the supernatants of the cell culture medium were analysed. Immunoblot analysis showed the presence of importin α1 in the supernatants of HCT116 and HepG2 cultures (Fig. 3a), whereas no importin α1 was detected in the supernatants of AGS (Fig. 3a) and A549 cultures (data not shown), showing that cell surface localization and extracellular release of importin α1 was correlated. Trypan blue exclusion assays indicated that the protein release was not due to a lack of cell integrity (cell viability >90%; data not shown). Additionally, no extracellular release of lamin A/C was detected (Fig. 3a). Thus, these results demonstrated that cancer cells release importin α1 into the extracellular space under normal culture conditions, and that this is correlated with its cell surface localization.

Next, to exclude the possibility that importin α1 in cultured medium was enclosed by a lipid layer, e.g., exosomes, we measured free importin α1 in cultured medium by a sandwich ELISA using two different anti-importin α1 antibodies. Under non-denatured conditions, free importin α1 was detected in the supernatant of HepG2 cultures, while the amount of importin α1 in the supernatant of AGS culture was comparable to background levels (Fig. 3b), indicating that cell surface importin α1-positive cancer cells release a free form of importin α1 into the extracellular milieu.

### Extracellular importin α1 associates with heparan sulfate on the cell surface

**Figure 4:**
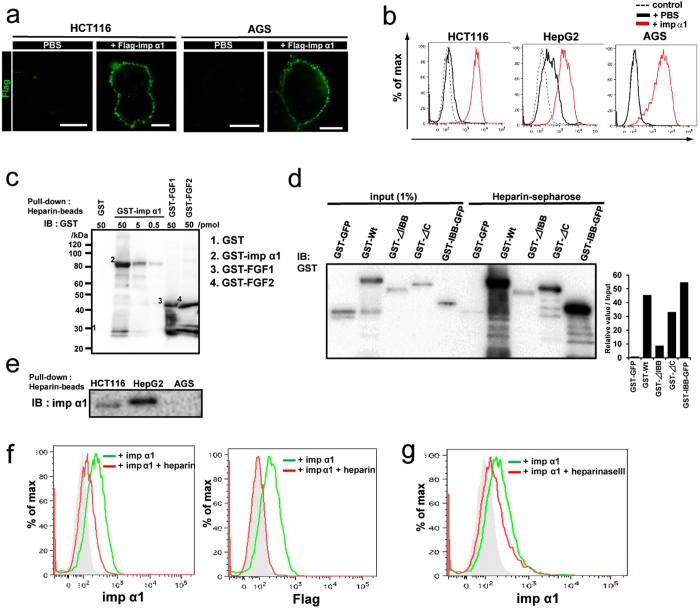
Extracellular importin α1 binds to the cell surface via heparan sulfate. (**a**) HCT116 or AGS cells were incubated in the presence of PBS or recombinant Flag-tagged importin α1 (100 ng/ml) on ice for 1 h. Flag staining was visualized using confocal laser microscopy. Scale bars: 10 μm. (**b**) Living cancer cell lines were treated with PBS or recombinant importin α1 (100 ng/ml). These cells were stained with anti-importin α1 antibody (Ab), and subjected to flow cytometric analysis. Dashed line, isotype control Ab. (**c**) GST or GST-tagged importin α1 at concentrations of 0.5, 5, or 50 pmol were immobilized on heparin-beads. The bound proteins were analysed using immunoblotting with anti-GST Ab. GST-tagged-FGF1 and -FGF2 (50 pmol) were used as positive controls. (**d**) GST-tagged importin α1 mutants, GST-GFP or IBB domain-fused GST-GFP at concentrations of 50 pmol were immobilized on heparin beads. The bound proteins were analysed using immunoblotting with anti-GST Ab. Band intensities of each protein bound to heparin-sepharose were normalized with that of each input sample using the Image J program. (**e**) Supernatants of cultured cancer cell lines were collected and immobilized on heparin-conjugated beads. Immunoblot analysis was performed using Abs to importin α1. (**f**) Living HCT116 cells were treated with recombinant Flag-tagged importin α1 (100 ng/ml) in the presence or absence of heparin (10 U/ml). These cells were stained with anti-importin α1 Ab (Left panel) or anti-Flag antibody (Right panel), and subjected to flow cytometric analysis. Gray area, isotype control Ab. (**g**) Living HCT116 cells were pretreated with or without heparinase III (0.2 U/ml) for 6 h, and then treated with recombinant Flag-tagged importin α1 (100 ng/ml). These cells were stained with anti-importin α1 antibody, and subjected to flow cytometric analysis. Gray area, isotype control Ab.

These findings led us to suppose that the importin α1 released into the extracellular space is attached to the cell surface. To verify this, we added recombinant Flag-importin α1 proteins to the cultured medium. As shown in Fig. 4a,b, we found apparent binding of the recombinant importin α1 to the cell surface of not only HCT116 cells and HepG2 cells, but also to that of AGS cells that were negative for cell surface localization of endogenous importin α1. These data indicated that importin α1 interacts with the cell membrane after its release into the extracellular space.

Next, we attempted to address how the released importin α1 binds to the cell surface. It is well known that many growth factors, such as fibroblast growth factors (FGFs), vascular endothelial growth factor (VEGF)-A and hepatocyte growth factor (HGF), have cationic amino-acid clusters^21^,^22^, and interact with heparan sulfate (HS), which includes heparin, which in turn is composed of one or more unbranched anionic polysaccharide(s) known as glycosaminoglycans (GAGs)^22^,^23^,^24^. Since importin α1 contains a hydrophilic cluster, including lysine and arginine residues, in its importin β binding (IBB) domain, conferring cationic properties to the protein, we hypothesized that importin α1 might be recruited to the cell surface through binding to HS. To assess this, we performed an *in vitro* solution-binding assay, in which recombinant importin α1 was mixed with heparin-conjugated beads. As expected, we found that heparin directly bound to importin α1 (Fig. 4c). To identify the domains of importin α1 important for binding to heparin, we prepared several mutants of importin α1, including an IBB domain-deleted mutant (ΔIBB) and a C-terminal region-deleted mutant (ΔC). The *in vitro* binding assay indicated that the association of the ΔIBB mutant with heparin was markedly weaker than that of the wild type and the ΔC mutant of importin α1 (Fig. 4d). In addition, we found that the IBB domain-fused GST-GFP proteins strongly bound to the heparin-beads (Fig. 4d), indicating that importin α1 was capable of associating with heparin via its IBB domain. In addition, when we applied heparin-beads to the cultured medium of cancer cells to determine whether the association between importin α1 and HS occurred in the extracellular space, rather than within cells, the heparin-conjugated beads significantly concentrated importin α1 in the cultured medium of HCT116, HepG2, PC9, and Hep3B cells, but not that of AGS and A549 cells (Fig. 4e and Supplementary Fig. S3).

To clarify the interaction between HS and importin α1 on the cell surface, HCT116 cells were incubated with recombinant importin α1 in the presence of free heparin. Association of importin α1 with the cells was clearly reduced by the addition of heparin (Fig. 4f). Moreover, when HCT116 cells were incubated with heparinase III, an enzyme that cleaves HS in cell surface proteoglycans, prior to importin α1 treatment^25^, we observed an apparent reduction of importin α1 binding to the cells (Fig. 4g). From these results, we concluded that importin α1 that has been released into the extracellular environment binds to HS on the cell surface.

### Cell surface importin α1 affects FGF1 signalling

**Figure 5:**
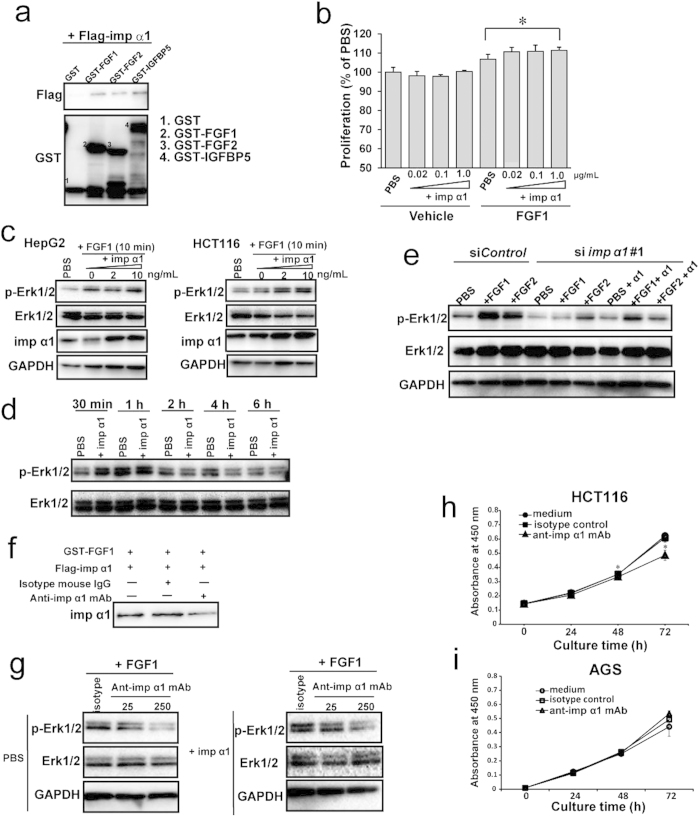
Extracellular importin α1 affects cell proliferation via interaction with FGF1 (**a**) GST, GST-FGF1, GST-FGF2, or GST-IGFBP5 (50 pmol) were incubated with 3 × Flag-importin α1 (50 pmol) immobilized on glutathione beads, and subjected to immunoblot analysis with the indicated antibodies. (**b**) Starved HCT116 cells were incubated in PBS or FGF1 (20 ng/ml) in the presence or absence of recombinant importin α1 at the indicated concentrations for 48 h. Cell proliferation was measured using Cell Counting Reagent. Data are means ± SD from three independent experiments. *P < 0.005. (**c**) The starved cells were incubated in PBS or FGF1 (20 ng/ml) in the presence or absence of recombinant importin α1 for 10 min, and subjected to immunoblot analysis with indicated antibodies. (**d**) Starved HCT116 cells were incubated in FGF1 (20 ng/ml) in the presence or absence of recombinant importin α1 (20 ng/ml) for the indicated time courses, and subjected to immunoblot analysis with indicated antibodies. (**e**) HCT116 cells were transfected with control or importin α1 siRNAs, followed by starvation and incubation in PBS or FGF1 or FGF2 (20 ng/ml) in the presence or absence of recombinant importin α1 (20 ng/ml) for 10 min, and subjected to immunoblot analysis with indicated antibodies. (**f**) GST-FGF1 (50 pmol) was incubated with 50 pmol 3 × Flag-importin α1 immobilized on glutathione beads in the presence of anti-importin α1 monoclonal antibody (mAb) or control mouse IgGs (as control), and subjected to immunoblot analysis. (**g**) Starved HCT116 cells were incubated in FGF1 (20 ng/ml) in the presence or absence of recombinant importin α1 (20 ng/ml) with anti-importin α1 mAb (25 or 250 ng/ml) or control mouse IgGs for 10 min, and subjected to immunoblot analysis with indicated antibodies. (**h**,**i**) Cell proliferation of HCT116 cells (**h**) and AGS cells (**i**) were measured for the indicated time courses after treatment without or with normal mouse IgGs as isotype control or anti-importin α1 mAb (250 ng/ml), in triplicate for each condition, using Cell Counting Reagent. Data are means ± SD from three independent experiments. *P < 0.005.

We next investigated which endogenous proteins associate with the cell surface importin α1. It has been reported that some secreted proteins, such as FGFs and epidermal growth factors (EGFs), are localized to and function in the nucleus, and that most of these proteins have their own cNLSs that are recognized by the importin α family and translocated into the nucleus^26^,^27^,^28^,^29^ (Supplementary Table S2). This raised the possibility that these proteins interact with the cell surface importin α1. Therefore, we selected candidates among these NLS-containing secreted proteins by ELISA screening. We found that the cNLS-containing secreted growth factors, such as FGF1, FGF2, insulin-like growth factor-binding protein (IGF-BP)3, and IGF-BP5, but not IFN-γ, bound to importin α1 (Supplementary Fig. S4). Furthermore, using an *in vitro* binding assay, we confirmed that recombinant GST-tagged FGF1, FGF2, and IGF-BP5 proteins interacted with importin α1 (Fig. 5a). In this study, we focused on FGF1 for further experiments.

We investigated whether the cell surface importin α1 affects FGF1 signalling in cancer cells. In HCT116 cells, the exogenous addition of FGF1 slightly increased cell proliferation (Fig. 5b). Under the same assay conditions, further addition of importin α1 slightly, but significantly, accelerated the proliferation of FGF1-stimulated HCT116 cells (Fig. 5b).

Next, to determine how the cell surface importin α1 is involved in the cellular response to FGF1, we monitored the activation of extracellular signal-regulated kinase 1/2 (ERK1/2), major targets of the FGF signalling pathway^30^,^31^,^32^. We found that the addition of importin α1 increased the phosphorylation of ERK1/2 in FGF1-stimulated HepG2 and HCT116 cells at an early stage (10 min) after stimulation (Fig. 5c), although this effect did not last up to late stages (1−6 h; Fig. 5d), meaning that importin α1 may function at the start of FGF1 signalling.

To confirm the involvement of the cell surface importin α1 in FGF1 signalling, we performed an RNAi study using HepG2 cells. Knockdown of importin α1 resulted in marked down-regulation of the activation of ERK1/2 in HepG2 cells in the presence of FGF1, and this suppressive effect was restored by the addition of exogenous importin α1 under the FGF1 stimulation (Fig. 5e). These results indicated that extracellular importin α1 affects FGF1 signalling on the cell surface.

To further confirm that importin α1 affects FGF1 signalling, we applied anti-importin α1 monoclonal antibody (mAb) to block the function of the extracellular and cell surface importin α1. We found that anti-importin α1 mAb impaired the interaction of importin α1 with FGF1 (Fig. 5f), and decreased the importin α1-mediated activation of ERK1/2 in response to FGF1 (Fig. 5g). These results indicated that the interaction of importin α1 with FGF1 plays an important role in the FGF1-mediated proliferative signalling (Fig. 5g).

Finally, we treated cancer cells with the anti-importin α1 mAb to establish whether the anti-importin α1 mAb can indeed diminish the proliferation of cancer cells. In HCT116 and HepG2 cells, cell proliferation was significantly reduced by anti-importin α1 mAb treatment (Fig. 5h and Supplementary Fig. S5), while the growth of AGS cells was not affected by the antibody (Fig. 5i). Taken together, these results demonstrated that the cell surface importin α1 plays crucial roles in the proliferation of colon cancer and hepatocellular carcinoma cells.

## Discussion

This study demonstrates the novel cellular localization and function of importin α1. That is, importin α1 is localized to and functions at the cell surface of some cancer cells. Indeed, we showed that cell surface importin α1 associates with growth factors, such as FGFs (FGF1 and FGF2), and enhances FGF1 signalling. Furthermore, we found that the treatment with an anti-importin α1 antibody impaired FGF1 signalling, resulting in the suppression of cancer cell proliferation.

Importin α1 has been regarded as a novel cancer marker in several cancers, because of its overexpression in some tumour tissues that correlates with tumour progression^14^,^15^,^18^,^33^. Hepatocellular carcinoma is one of the cancers that show an aberrant expression of importin α1^14^. Decreased proliferation of hepatocellular carcinoma cell lines after knockdown of importin α1 has been shown in a previous report^14^. Our present study showed that importin α1 is detected at relatively higher levels at the cell surface of at least three hepatocellular carcinoma cell lines (HepG2, Hep3B, and HLE) than that of other types of cancer cell lines. Moreover, we found that cell growth was suppressed when the HepG2 cells were treated with a monoclonal antibody specific for importin α1. Therefore, these results suggested that importin α1 localized to the cell surface is involved in the cell growth of at least some hepatocellular carcinomas.

Several studies have so far reported that some proteins that were originally identified as having a cytoplasmic or nuclear localization, such as calreticulin (endoplasmic reticulum, ER), GRP78/BiP (ER), hnRNP-K (nucleus), and nucleolin (nucleus)^19^,^34^,^35^,^36^,^37^,^38^,^39^, can be expressed on the cell surface. Furthermore, it has been shown that most of these are translocated to the cell surface in response to cellular stresses, such as ER stress (GRP78/BiP) and cold stimulation (hnRNP-K). On the other hand, we showed that importin α1 was expressed on the cell surface of some cancer cells under physiological conditions, similar to nucleolin^39^. Furthermore, our data revealed that although the total protein levels of importin α1 in AGS cells was comparable to those in HCT116 cells and HepG2 cells, importin α1 was not detected on the cell surface of AGS cells. These results suggested a novel intracellular trafficking, by which soluble intracellular proteins are transported to the cell surface and released from within cells in a cell condition- and/or cell type-dependent manner.

It has recently been reported that importin α1 is present in the sera of lung cancer patients^18^. Consistently, we also found that importin α1 was detected in culture media of several cancer cell lines. Furthermore, we found that exogenously added importin α1 was associated with the cell surface and bound to HS on the cell surface. These results suggested that the released importin α1 binds to the cell surface of adjacent cells via HS in tumour tissues.

It is known that FGFs can regulate cellular functions through an evolutionarily conserved signalling module that functions in both invertebrates and vertebrates^40^. The FGF family proteins, including the prototype members FGF1 and FGF2, are potent regulators of cell proliferation, differentiation, migration, and survival. On the cell surface, FGFs bind to two types of receptors; the high-affinity tyrosine kinase FGF receptors (FGFRs) and the low-affinity HS proteoglycan (HSPG). Activated FGFR initiates downstream signalling cascades, such as the pathways involving Ras/MAPK, phosphoinisitide 3-kinase (PI3K)/AKT, and phosholipase Cγ/PKC. The ERK signalling cascade is reported to control the proliferation of multiple cell types in response to growth factor treatment^30^. On the other hand, HSPG serves to recruit FGFs to FGFRs, and to stabilize the FGF−FGFR axis to maintain their downstream signalling^21^. In this study, we showed that importin α1 interacts with both FGFs and HS. Furthermore, we found that the addition of importin α1 recombinant proteins to culture cells enhanced the phosphorylation of ERK1/2 at the initial step of FGF signalling after FGF1 stimulation. Taken together, we propose the following model. (1) Importin α1 is released from within cells and the released importin α1 binds to the cell surface via HS. (2) The cell surface importin α1 helps to recruit FGF, to accelerate FGF signalling at the initial step. Thus, it is biologically important to know how importin α1 is transported to the cell surface and released from within cells.

It is also known that FGFs have cNLSs and accumulate in the nucleus in an importin α/β-dependent manner. However, it is still unknown how extracellular FGFs traverse the cell membrane to enter the cell. Thus, it is interesting to know whether cell surface importin α1 is also involved in the internalization of extracellular FGFs. Further studies will be required to address these questions.

Cell surface proteins specific to cancer cells and soluble proteins in serum that promote tumorigenesis have been considered as potential targets for the treatment of cancers. Therapies targeted at the extracellular importin α1 (both the released form and the cell surface-localized form), as well as those that are targeted at released importin α1-mediated induction of FGF1 signalling, are expected to perturb the proliferation of importin α1-releasing cancer cells, thereby providing a novel option for the treatment of cell surface importin α1-related cancers.

## Methods

### Cell Culture

The colon cancer line HCT116, the hepatocellular carcinoma lines HepG2, Hep3B, and HLE, the lung cancer lines A549 and PC9, the breast cancer lines MCF-7, and MRK-nu-1, the fibroblast cell line TIG-1, and the gastric cancer lines KATOIII and AGS were obtained from the Japanese Collection of Research Bioresources (Osaka, Japan). The breast cancer lines, SKBr3 and MDA-MB-231, and the normal mammary epithelial cell line MCF-10A, were kindly provided by H. Fujii (National Cancer Center Hospital East, Chiba, Japan). HCT116, HepG2, Hep3B, HLE, TIG-1, A549, and SKBr3 were cultured in Dulbecco’s modified Eagle’s medium (DMEM; Sigma, St. Louis, MO, USA) supplemented with 10% fetal bovine serum (FBS; Sigma). KATOIII, AGS, PC9, and MDA-MB-231 were cultured in RPMI1640 (Sigma) supplemented with 10% FBS. The microvascular endothelial cells dHMVEC (Bio Whittaker, Walkersville, MD, USA) was cultured in growth factor complete endothelial basal media (EBM; Lonza, Walkersville, MD, USA) supplemented with 5% FBS. MCF-10A was cultured in MEGM BulleKit (Lonza). MCF7 was cultured in MEM (Sigma) supplemented with 0.1 mM NEAA, 1 mM sodium pyruvate, 10 μg/mL insulin, and 10% FBS. The breast cancer line MRK-nu-1 was cultured in DMEM/F12 (Sigma) supplemented with 10% FBS.

### Recombinant proteins

Recombinant EGF, FGF1, FGF2, IGF-BP3, IGF-BP5, and IFN-γ1 were purchased from Peprotech (Rocky Hill, NJ, USA). pGEX-6P2-3 × Flag-importin α1 (human) was obtained as described previously^8^. pGEX-2T constructs (GST-GFP, GST-NLS-GFP, and GST-IBB-GFP) were obtained as described previously^20^. The mouse importin α1 deletion mutants (encoding either amino acids 61−529 [ΔIBB domain] or 1−500 [ΔC-terminus]) were constructed using a KOD-plus-Mutagenesis Kit (Toyobo, Tokyo, Japan)^41^. The mutants were cloned into pGEX-6P-2 vector, which is an expression vector for the production of glutathione S-transferase (GST)-fused protein. Transformation of expression vectors, protein expression, bacterial lysis, and purification of the proteins were performed as described previously^8^,^42^. GST was cleaved using PreScission protease (GE Healthcare, Piscataway, NJ, USA) in cleavage buffer (50 mM Tri-HCL [pH 7.5], 150 mM NaCl, 1 mM EDTA[3Na], 1 mM dithiothreitol [DTT], containing 1 μg/mL each of aprotinin, leupeptin, and pepstatin).

### Pull-down assay

The proteins were added to transport buffer (20 mM HEPES/NaOH, pH 7.4, 110 mM potassium acetate, 2 mM magnesium acetate, 5 mM sodium acetate, 0.5 mM EGTA/NaOH, 2 mM DTT) in the presence or absence of anti-importin α1 mAb, and mixed with glutathione-sepharose 4B beads (GSH-beads, GE Healthcare) or heparin-sepharose beads (Sigma). The mixtures were incubated at 4 °C for 1 h and then washed with transport buffer. Bound proteins were eluted with sample buffer for sodium dodecylsulfate-polyacrylamide gel electrophoresis (SDS-PAGE).

### Plasmids and siRNA transfection

p3 × Flag-CMV-importin α1 and p3 × Flag-CMV empty vectors were transfected into HCT116 cells with Lipofectamine 3000 (Thermo Fisher Scientific, Rockford, IL, USA). The sequences of the siRNA (chimeric RNA-DNA) duplexes have previously been described^43^. Cells were transfected with each siRNA for 48 h using Oligofectamine reagent (Thermo Fisher Scientific).

### Flow cytometry

Cells were dissociated by Accutase (Thermo Fisher Scientific), and pelleted by centrifugation at 500 × *g* for 5 min at 4 °C. Cell suspensions were incubated with anti-human importin α1 mouse mAb (1:200 dilution; BD Biosciences, San Diego, CA, USA), Alexa488-conjugated anti-mouse IgGs (1:200 dilution; Thermo Fisher Scientific) in phosphate-buffered saline (PBS) containing 0.1% FBS. Suspensions were incubated for 30 min at 4 °C. Flow cytometric analysis was performed using FACSCantoII (Becton Dickinson, Flanklin Lakes, NJ, USA). At least three independent experiments were performed.

### Cell surface biotinylation and immunoblotting

Cells were washed twice with PBS containing 1 mM CaCl_2_ and 0.5 mM MgCl_2_ (PBS-CM) at 4 °C, and incubated for 15 min at 4 °C in 1.0 mg/mL of sulfo-N-hydroxysulfosuccinimide (sulfo-NHS)-Biotin (Thermo Fisher Scientific) dissolved in PBS-CM. To quench unreacted biotin, cells were washed three times with PBS-CM plus 50 mM glycine and twice with PBS-CM^44^. Cell surface biotinylated cells were lysed by RIPA buffer (50 mM Tris/HCl, pH 7.5, 150 mM NaCl, 1.25% NP-40, 0.1% SDS, 0.5% Deoxycholic acid). Protein concentrations of each fraction were determined using the BCA protein assay kit (Thermo Fisher Scientific). Cell lysates were bound directly to Neutravidin beads for 3 h at 4 °C. The beads were washed five times with NET-2 buffer (250 mM Tris-HCl [pH7.5], 750 mM NaCl, 0.25% Nonidet P-40) and then subjected to immunoblot analysis^45^. Proteins were subjected to SDS-PAGE and transferred to a polyvinylidene fluoride (PVDF) membrane. The membranes were probed with the following antibodies: anti-importin α1 mouse mAb (1:1,000 dilution; BD Biosciences), anti-importin β1 mouse mAb (1:1,000 dilution; BD Bioscience), anti-GAPDH rabbit polyclonal antibody (pAb; 1:1,000 dilution; Sigma), anti-phospho-Erk1/2 rabbit pAb (1:1,000 dilution; CST), anti-Erk1/2 rabbit pAb (1:1,000 dilution; CST, Japan), anti-LaminA/C rabbit pAb (1:1,000 dilution; CST, Tokyo, Japan), anti-Flag M2 mouse mAb (1:1,000 dilution; Sigma). Signals were detected by enhanced chemiluminescence (ECL, Thermo Fisher Scientific).

### Sandwich enzyme-linked immunosorbent assay (ELISA)

ELISA was performed using MaxiSorp plate (Thermo Fisher Scientific) coated with 0.5 μg/well of recombinant proteins. Candidate proteins or conditioned medium were incubated with plate-bound each protein for 1 h. The antigen-antibody complexes were detected with 1:5,000-diluted HRP-conjugated second antibodies with 3, 3′, 5, 5′-tetramethylbenzidine (TMB; Dako Cytomation, Glostrup, Denmark) as the substrate. OD was read at 450 nm using a Bio-Rad Microplate Reader Model 680 (Bio-Rad Laboratories, Hercules, CA, USA).

### Immunofluorescence analysis

Cultured cells grown on glass coverslides were fixed in 4% paraformaldehyde for 15 min at room temperature; then, cells were permeabilized with PBS containing 0.1% Triton-X100 and 1 mg/mL bovine serum albumin (BSA) for 5 min. The cells were washed three times with PBS before incubation at 4 °C overnight with anti-importin α1 mouse mAb (1:200 dilution; BD Bioscience). After washing three times with PBS, the cells were incubated at room temperature for 30 min with the appropriate Alexa Fluor 488-, or 647-conjugated secondary antibodies and stained with Hoechst 33342 (Thermo Fisher Scientific) for detection of nuclei. Cells were observed by confocal fluorescence microscopy (TCS SP8, Leica, Mannheim, Germany).

### Proliferation assay

Cells were cultured in 96-well plates either in media alone, with isotype control IgG or anti-importin α1 mAb (250 ng/mL; BD Biosciences) in a total volume of 100 μL (3 × 10^3^ cells/well). After 24, 48, and 72 h of incubation at 37 °C, 2-(2-methoxy-4-nitrophenyl)-3-(4-nitrophenyl)-5-(2,4-disulfophenyl)-2H-tetrazolium (Nacalai Tesque Inc, Tokyo, Japan) was added to each well. After 1 h of incubation, the water-soluble formazan dye, 1-methoxy-5-methylphenazinium, which is formed upon bio-reduction in the presence of an electron carrier, was measured in a microplate reader (Bio-Rad) at 450 nm. All samples were assayed in triplicate, and the results reported were means of triplicate wells.

### Statistical analysis

Data are presented means ± SD and were assessed for statistical significance using the unpaired Student’s t test.

## Additional Information

**How to cite this article**: Yamada, K. *et al.* Cell surface localization of importin α1/KPNA2 affects cancer cell proliferation by regulating FGF1 signalling. *Sci. Rep.* **6**, 21410; doi: 10.1038/srep21410 (2016).

## Supplementary Material

Supplementary Information
