# Supplementary material for: Cell surface localization of importin α1/KPNA2 affects cancer cell proliferation by regulating FGF1 signalling
Source: Sci Rep. 2016 Feb 18;6:21410. doi: 10.1038/srep21410 (PMC4757827; doi:10.1038/srep21410)
Supplement: Supplementary Information [file srep21410-s1.doc]

**Supporting Information**

**Cell surface localization of importin α1/KPNA2 affects cancer cell proliferation by regulating FGF1 signalling**

Kohji Yamada^1^, Yoichi Miyamoto^1^, Akira Tsujii^1,2^, Tetsuji Moriyama^1^, Yudai Ikuno^1^, Takashi Shiromizu^3^, Satoshi Serada^4^, Minoru Fujimoto^4^, Takeshi Tomonaga^3^, Tetsuji Naka^4^, Yoshihiro Yoneda^5*^, & Masahiro Oka^1*^

^1^Laboratory of Nuclear Transport Dynamics, National Institutes of Biomedical Innovation, Health and Nutrition, 7-6-8 Saito-Asagi, Ibaraki, Osaka 567-0085, Japan

^2^Department of Genetics, Graduate School of Medicine, Osaka University, 1-3 Yamadaoka, Suita, Osaka 565-0871, Japan

^3^Laboratory of Proteome Research, National Institutes of Biomedical Innovation, Health and Nutrition, 7-6-8 Saito-Asagi, Ibaraki, Osaka 567-0085, Japan

^4^Laboratory of Immune Signal, National Institutes of Biomedical Innovation, Health and Nutrition, 7-6-8 Saito-Asagi, Ibaraki, Osaka 567-0085, Japan

^5^National Institutes of Biomedical Innovation, Health and Nutrition, 7-6-8 Saito-Asagi, Ibaraki, Osaka 567-0085, Japan

^*^Corresponding authors

Yoshihiro Yoneda

National Institutes of Biomedical Innovation, Health and Nutrition,

7-6-8 Saito-Asagi, Ibaraki, Osaka 567-0085, Japan

Phone: +81 72-641-9012

FAX: +81 72-641-9013

E-mail: y-yoneda@nibiohn.go.jp

Masahiro Oka

Laboratory of Nuclear Transport Dynamics, National Institutes of Biomedical Innovation, Health and Nutrition,

7-6-8 Saito-Asagi, Ibaraki, Osaka 567-0085, Japan

Phone: +81 72-641-9012

FAX: +81 72-641-9013

E-mail: [moka@nibiohn.go.jp](mailto:moka@nibiohn.go.jp)

Supplementary Figures S1-S5

Supplementary Table S1-S2

Supplementary Methods

Supplementary References

Table S1.

**Supplementary Fig. S1.** **Cell surface expression of importin α1 in cancer cell lines.** Expression of importin α1 on the cell surface of living human cancer cell lines. Blue line, goat anti-importin α1 antibody (Ab; C-20, Santa Cruz, Dallas, TX, USA). Gray area, isotype control Ab.

**Supplementary Fig. S2.** **Knockdown of importin α1 in HCT116 cells.** (**a**) Living HCT116 cells transfected with control or importin α1 siRNAs were lysed, and subjected to immunoblotting analysis.

Living HCT116 cells transfected with control or importin α1 siRNAs were stained with importin α1 with (**b**) or without (**c**) permeabilization by Triton X-100, and subjected to flow cytometric analysis (right panel). Data are normalized to control, and are presented as mean ± SD values from three independent experiments (left panel).

**Supplementary Fig. S3.** **Detection of importin α1 in conditioned medium of cancer cell lines.** Supernatants of each culture cell line were immobilized on heparin beads (20 μl). The bound proteins were analysed using immunoblotting with anti-importin α1 antibody.

**Supplementary Table S2**

NLS mapper; <http://nls-mapper.iab.keio.ac.jp/cgi-bin/NLS_Mapper_form.cgi>

PSORTII; http://psort.hgc.jp/form2.html

**Supplementary Fig. S4. ELISA screening for binding partners of importin α1** Binding activity of each candidate with importin α1 was examined by ELISA analysis. Plates were coated with each recombinant protein of candidate (**a**) or recombinant importin α1 (**b** and **c**), and incubated with additional recombinant importin α1 (**a**), recombinant FGF1 (**b**), or recombinant FGF2 (**c**). The primary antibodies used were as follows: anti-importin α1 (**a**), anti-FGF1 (**b**), or anti-FGF2 (**c**).

**Supplementary Fig. S5.** **Inhibition of cell growth by anti-importin α1 in HepG2 cells.** HepG2 cells were seeded at a density of 3 × 10^3^ cells/well in 96-well plates. Cell proliferation was measured for the indicated times after treatment without or with normal mouse IgGs as isotype control or anti-importin α1 mAb (250 ng/ml), in triplicate for each condition, using Cell Counting Reagent. Data are means ± SD from three independent experiments. * *P* < 0.005.

**Supplementary Methods**

**Material and method**

**Colorectal cancer tissue and cell culture samples.** 12 of colorectal cancer (CRC) tissue samples were obtained from Chiba University School of Medicine. Tissue samples were surgically resected, and then frozen in liquid nitrogen and stored at −80 °C until further analyses. Informed consent was obtained from all donors, and the protocol was approved by the ethics committees of the National Institute of Biomedical Innovation Health and Nutrition, and the Chiba University School of Medicine. 5 human colorectal carcinoma cell lines HCT116 (ATCC; CCL-247), DLD-1 (ATCC; CCL-221), RKO (ATCC; CRL-2577), SW480 (ATCC; CCL-228) and SW620 (ATCC; CCL-227) were grown in RPMI-1640 (Gibco Laboratories) medium with 10% fetal bovine serum and antibiotics. Cells were maintained at 37°C in an incubator supplemented with 5% CO_2_ until they grew to sub-confluence.

**Membrane protein extraction and digestion.** Extraction of membrane protein was described in previous work [^1^](#_ENREF_1). Tissue and cell culture samples were washed with ice-cold PBS and then homogenized with a dounce homogenizer in ice-cold PBS containing a protease inhibitor cocktail (Roche Diagnostics, Mannheim, Germany). The homogenate was centrifuged at 1000×g for 10 min at 4 °C, and the post-nuclear supernatant was centrifuged at 100,000×g for 1 h at 4 °C. The pellet was suspended in ice-cold 0.1 M Na_2_CO_3_ solution and centrifuged at 100,000×g for 1 h at 4 °C. After centrifugation, the pellet was solubilized by MPEX PTS reagent kit (GL Science, Tokyo, Japan) at 95 °C for 5 min followed by sonication for 5 min and centrifugation at 100,000×g for 30 min at 4 °C. The supernatant was contained as membrane fraction extract. Protein concentration was quantified by using a DC protein assay kit (Bio-Rad, Hercules, CA, USA). The pooled sample of tissue and cultured cell were prepared by mixing an equal amount of each membrane extracts. Proteolytic digestion were performed using a phase transfer surfactant (PTS) protocol[^2^](#_ENREF_2). Briefly, 20μg of pooled proteins were reduced with 1/20 volume of 100mM DTT in 50mM NaHCO_3_ for 30 min at room temperature (RT). For alkylation, 1/20 volume of 550 mM iodoacetic acid in 50 mM NaHCO_3_ was added and incubated for 30 min at RT. And then, sample was digested with 1% (w/w) of trypsin (proteomics grade; Roche Mannheim, Germany) for 12 hours at 37°C. After digestion, an equal volume of ethyl acetate was added to digested samples, the mixtures were acidified by 1% trifluoroacetic acid, and vortexed to transfer detergents to the organic phase. After centrifugation, the aqueous phase containing peptides was collected and desalted by using C18 Stage Tip [^3^](#_ENREF_3)s

**LC-MS/MS and proteomic data analysis.** Digested peptides were analyzed by Q-Exactive mass spectrometer (Thermo Scientific, Bremen, Germany) with UltiMate 3000 Nano LC system (Thermo Scientific, Bremen, Germany) and a HTC-PAL autosampler (CTC Analytics, Zwingen, Switzerland). The analytical column were packed an reverse-phase material ReproSil-Pur C18-AQ, 1.9 μm resin (Dr. Maisch, Ammerbuch-Entringen, Germany) into a self-pulled needle (300 mm length × 75 μm inner diameter). The mobile phases consisted of buffer A (0.1% formic acid and 2% acetonitrile) and B (0.1% formic acid and 90% acetonitrile). 2 μg of digested peptides were dissolved in buffer A and loaded onto a trap column (0.075 × 20 mm, Acclaim PepMap RSLC Nano-Trap Column; Thermo Scientific). The nanoLC gradient was delivered at 280nL/min and consisted of a linear gradient of buffer B developed from 5 to 35% B in 120 min. Full MS scans were performed using an orbitrap mass analyzer (scan range 350-1800 m/z, with a resolution of 70 000 after accumulation of ions to a 3×10^6^ target value). The 10 most intense precursor ions were selected and fragmented in the octopole collision cell by higher-energy collisional dissociation (HCD) with a maximum injection time of 120 ms and a resolution of 35 000. The MS/MS ion selection threshold was set to 5 × 10^4^ counts. A 3.0 Da isolation width was chosen. The HCD raw data were extracted and searched separately against UniProt human database (release 2011_11), using Proteome Discoverer 1.3 (Thermo Scientific) and Mascot v2.3. The precursor mass tolerance was set to 7 ppm, and fragment ion mass tolerance was set to 0.01 Da. The search parameters allowed two missed cleavage for trypsin, fixed modifications (carbamidomethylation at cysteine), and variable modifications (oxidation at methionine). Peptides and proteins were accepted with a false discovery rate of <1%, which was estimated on the basis of the number of accepted hits from the reverse database.

**Recombinant proteins.** Recombinant EGF, FGF1, FGF2, IGF-BP3, IGF-BP5, and IFN-γ1 were purchased from Peprotech (Rocky Hill, NJ, USA). pGEX-6P2-3×Flag-importin α1 (human) was obtained as described previously[^4^](#_ENREF_4). Transformation of expression vectors, protein expression, bacterial lysis, and purification of the proteins were performed as described previously. GST was cleaved using PreScission protease (GE Healthcare, Piscataway, NJ, USA) in cleavage buffer (50 mM Tri-HCL [pH 7.5], 150 mM NaCl, 1 mM EDTA[3Na], 1 mM dithiothreitol [DTT], containing 1 µg/mL each of aprotinin, leupeptin, and pepstatin).

**Pull-down assay.** The proteins were added to transport buffer (20 mM HEPES/NaOH, pH 7.4, 110 mM potassium acetate, 2 mM magnesium acetate, 5 mM sodium acetate, 0.5 mM EGTA/NaOH, 2 mM DTT) in the presence or absence of anti-importin α1 mAb, and mixed with glutathione-sepharose 4B beads (GSH-beads, GE Healthcare) or heparin-sepharose beads (Sigma, St. Louis, MO, USA). The mixtures were incubated at 4°C for 1 h and then washed with transport buffer. Bound proteins were eluted with sample buffer for sodium dodecylsulfate-polyacrylamide gel electrophoresis (SDS-PAGE).

**siRNA transfection.** The sequences of the siRNA (chimeric RNA-DNA) duplexes have previously been described[^6^](#_ENREF_6). Cells were transfected with each siRNA for 48 h using Oligofectamine reagent (Thermo Fisher Scientific, Rockford, IL, USA).

**Flow cytometry.** Cells were dissociated by Accutase (Thermo Fisher Scientific), and pelleted by centrifugation at 500 × *g* for 5 min at 4°C. Cell suspensions were incubated with anti-human importin α1 mouse mAb (1:200 dilution; BD Bioscience, San Diego, CA, USA), Alexa488-conjugated anti-mouse IgGs (1:200 dilution; Thermo Fisher Scientific), Alexa488-conjugated streptavidin (1:200 dilution; Thermo Fisher Scientific) in phosphate-buffered saline (PBS) containing 0.1% FBS. Suspensions were incubated for 30 min at 4°C. Flow cytometric analysis was performed using FACSCantoII (Becton Dickinson, Flanklin Lakes, NJ, USA). At least three independent experiments were performed.

**Sandwich enzyme-linked immunosorbent assay (ELISA).** ELISA was performed using MaxiSorp plate (Thermo Fisher Scientific) coated with 0.5 μg/well of recombinant proteins. Candidate proteins or conditioned medium were incubated with plate-bound each protein for 1 h. The antigen-antibody complexes were detected with 1:5,000-diluted HRP-conjugated second antibodies with 3, 3′, 5, 5′-tetramethylbenzidine (TMB; Dako Cytomation, Glostrup, Denmark) as the substrate. OD was read at 450 nm a Bio-Rad Microplate Reader Model 680 (Bio-Rad Laboratories, Hercules, CA, USA).

**Proliferation assay.** Cells were cultured in 96-well plates either in media alone, with isotype control IgG or anti-importin α1 mAb (250 ng/mL; BD Bioscience) in a total volume of 100 μL (3 × 10^3^ cells/well). After 24, 48, and 72 h of incubation at 37°C, 2-(2-methoxy-4-nitrophenyl)-3-(4-nitrophenyl)-5-(2,4-disulfophenyl)-2H-tetrazolium (Nacalai Tesque Inc, Tokyo, Japan) was added to each well. After 1 h of incubation, the water-soluble formazan dye, 1-methoxy-5-methylphenazinium, which is formed upon bio-reduction in the presence of an electron carrier, was measured in a microplate reader (Bio-Rad) at 450 nm. All samples were assayed in triplicate, and the results reported were means of triplicate wells.

**Statistical analysis.** Data are presented means ± SD and were assessed for statistical significance using the unpaired Student’s t test.

**Supplementary References**

1. Kume, H.*, et al.* Discovery of colorectal cancer biomarker candidates by membrane proteomic analysis and subsequent verification using selected reaction monitoring (SRM) and tissue microarray (TMA) analysis. *Mol. Cell. Proteomics* **13**, 1471-1484 (2014).

2. Masuda, T., Tomita, M. & Ishihama, Y. Phase transfer surfactant-aided trypsin digestion for membrane proteome analysis. *J. Proteome Res.* **7**, 731-740 (2008).

3. Rappsilber, J., Mann, M. & Ishihama, Y. Protocol for micro-purification, enrichment, pre-fractionation and storage of peptides for proteomics using StageTips. *Nat. Protoc.* **2**, 1896-1906 (2007).

4. Kimoto, C.*, et al.* Functional characterization of importin alpha8 as a classical nuclear localization signal receptor. *Biochim. Biophys. Acta* (2015).

5. Imamoto, N.*, et al.* In vivo evidence for involvement of a 58 kDa component of nuclear pore-targeting complex in nuclear protein import. *EMBO J.* **14**, 3617-3626 (1995).

6. Fukumoto, M., Sekimoto, T. & Yoneda, Y. Proteomic analysis of importin alpha-interacting proteins in adult mouse brain. *Cell Struct. Funct.* **36**, 57-67 (2011).
